# Revision of the Original Material of *Plagiothecium denticulatum* var. *obtusifolium* (Turn.) Moore and New Synonyms for This Taxon

**DOI:** 10.3390/plants11192446

**Published:** 2022-09-20

**Authors:** Grzegorz J. Wolski, Daniel Bożyk, Jarosław Proćków

**Affiliations:** 1 Department of Geobotany and Plant Ecology, Faculty of Biology and Environmental Protection, University of Lodz, ul. Banacha 12/16, 90-237 Łódź, Poland; 2 Department of Plant Biology, Institute of Environmental Biology, Wrocław University of Environmental and Life Sciences, ul. Kożuchowska 7a, 51-631 Wrocław, Poland

**Keywords:** *Plagiothecium denticulatum* var. *obtusifolium*, *P. denticulatum* var. *auritum*, *P. sandbergii*, synonymization, typification

## Abstract

*Hypnum denticulatum* var. *obtusifolium* was described by Turner in the early nineteenth century. This taxon, now known as *Plagiothecium denticulatum* var. *obtusifolium*, has not been studied in detail or received a detailed description. During the revision in the Natural History Museum (herbarium BM), a specimen described there as a type (BM000890810) was found, but a careful analysis showed that it is currently not a type specimen. On the other hand, the conducted research showed that the holotype of this taxon is the figure attached by Turner to the newly described taxon. However, because the holotype does not contain all taxonomically significant features, the specimen found was used to designate the epitype of this name (BM000890810). Therefore, this paper provides the first complete description of the qualitative and quantitative characteristics of this taxon, which is today known as *P*. *denticulatum* var. *obtusifolium*. Forty qualitative and quantitative characteristics analyzed made it possible to make redescription of the examined taxon. Moreover, analysis of two morphologically similar, currently distinct taxa, *P. sandbergii* and *P*. *denticulatum* var. *auritum*, revealed no differences between them. Therefore, these taxa were proposed as new synonyms of *P*. *denticulatum* var. *obtusifolium*. Additionally, for both taxa mentioned above, lectotypes were proposed for *P*. *denticulatum* var. *auritum*, specimen PC0132639, and for *P*. *sandbergii*, specimen PC0132604, both from the Muséum National d’Histoire Naturelle, Paris, France (Herbarium PC).

## 1. Introduction

The history of *Plagiothecium denticulatum* var. *obtusifolium* (Turner) Moore, initially named *Hypnum denticulatum* var. *obtusifolium* Turner, began in 1804, when the English botanist D. Turner examined material collected by D. Brown on the summit of Mount Bulbein, in the town of Donegal, located on the Inishowen Peninsula in Northern Ireland [1].

**Figure 1:**
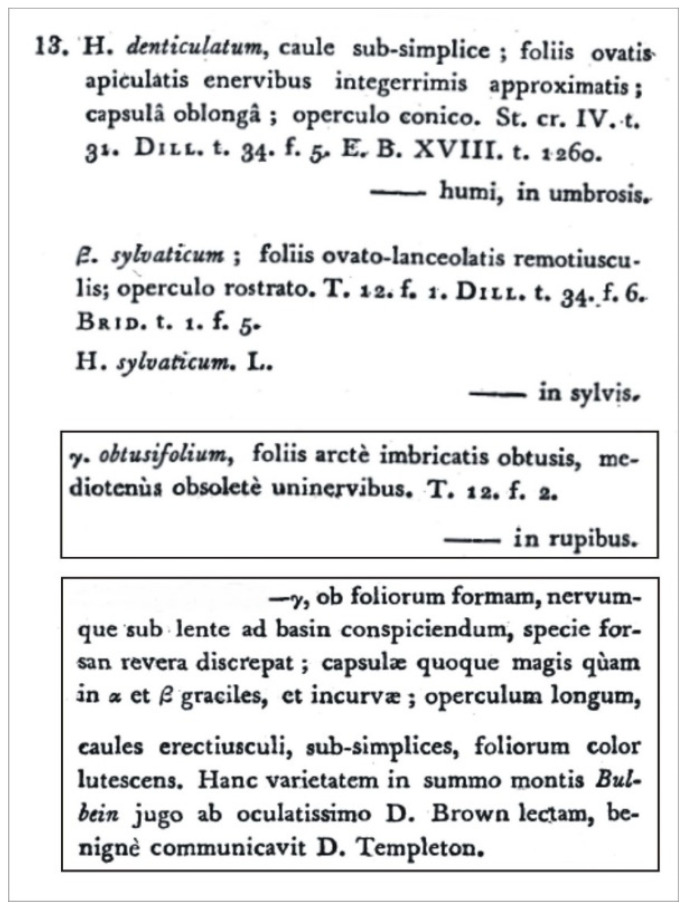
Diagnosis of *Hypnum denticulatum* var. *obtusifolium* [1]. Fragments concerning the described taxon are marked in the frame.

**Figure 2:**
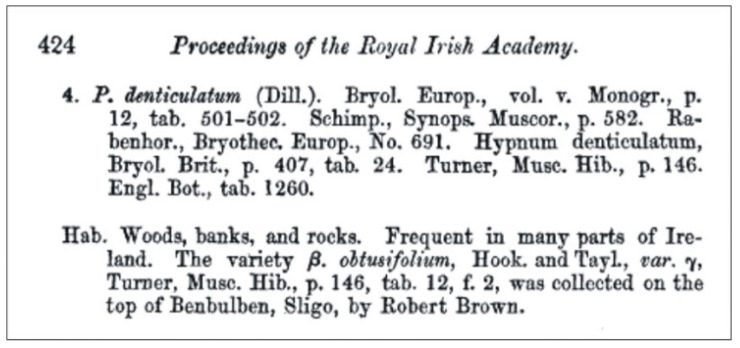
Description of *Plagiothecium denticulatum* var. *obtusifolium* [2].

Turner [1] observed that the capsules of this specimen were more slender and curved than those of the typical *Hypnum denticulatum* Hedw. (=*P*. *denticulatum* (Hedw.) Schimp.) or *H*. *denticulatum* var. *sylvaticum* (Brid.) Turn. For this reason, the author decided to distinguish this taxon as a separate variety, as *H*. *denticulatum* var. *obtusifolium* (Figure 1), by publishing its description in *Muscologiae Hibernicae Spicilegium* [1]. Sixty-nine years later, *H*. *denticulatum* var. *obtusifolium* was incorporated into the genus *Plagiothecium* Schimp. by D. Moore. As a result, the name was changed to *P*. *denticulatum* var. *obtusifolium* (Turner) Moore [2], and this remains the accepted name today (Figure 2).

**Figure 3:**
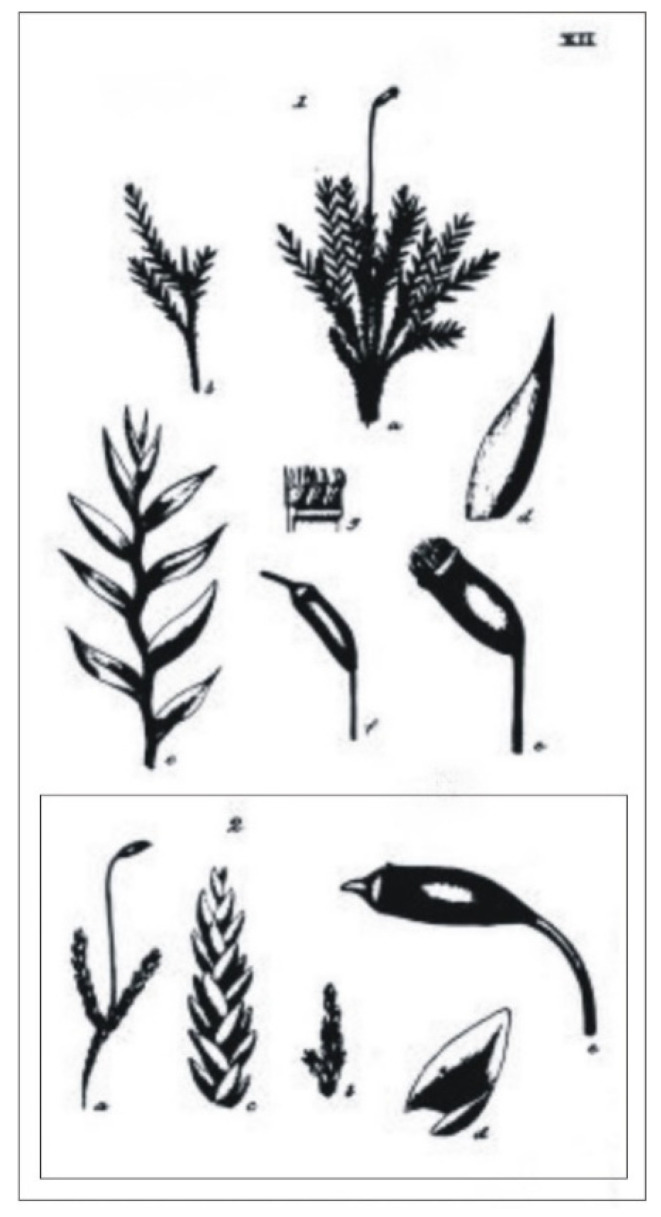
Original graphics from Turner’s manuscript [1]. Explanation: *H*. *denticulatum* is given in the upper part and *H*. *denticulatum* var. *obtusifolium* at the bottom (in the frame). Downloaded from https://archive.org (accessed on 15 April 2022).

In the diagnosis of a new taxon, Turner [1] did not cite any specimen or collection as a type. He only referred to the figure “T. 12. f. 2.”, which showed the differences between the current *H*. *denticulatum* and the newly described *H*. *denticulatum* var. *obtusifolium* (Figure 3).

*Plagiothecium denticulatum* var. *obtusifolium* was not distinguished for more than 200 years. The name has been commonly applied to specimens of *P*. *denticulatum* that were characterized by acute to obtuse leaves and well-developed ligulate decurrencies [3]. In addition, over the two centuries since, many authors have described dozens of subspecies, varieties, and forms. This has resulted in the appearance of many synonyms that function at the level of varieties, which have sometimes even been raised to the rank of species [3,4,5,6,7,8,9,10,11,12].

For example, Smith [4] published a new taxon under the name *H*. *donnianum* Sm. A few years later, Wahlenberg [5] published the name *H*. *obtusatum* Wahl. describing the specimen as “*surculis subfasciculatis simplicibus planis, foliis ovalibus obtusis distichis subenervibus, capsulis cylindraceis subcernuis*”, indicating that this specimen is characterized by simple flattened young shoots; oval, obtuse leaves, with slender alternate, imbricate foliage; and cylindrical capsules. Six years later, Hooker and Taylor [6] recognized the two species mentioned above as synonyms of *H*. *denticulatum* var. *obtusifolium* (=*P. denticulatum* var. *obtusifolium*).

In the following decades, more names based on Smith’s *H*. *donnianum*, at different ranks, were combined: *P*. *donnianum* (Sm.) Mitt.; *P*. *denticulatum* subsp. *donnianum* (Sm.) Giac., *H*. *denticulatum* var. *donnianum* (Sm.) Hook.; and *P*. *denticulatum* var. *donnianum* (Sm.) Lindb. ex Weim. Since the authors mentioned above [6,8,9,11,13] based their names on *H*. *donnianum* Sm., the epithet “*donii*” is most likely to be an orthographic equivalent of *donnianum*.

On the other hand, Bridel [7] proposed to change the rank of *H*. *denticulatum* var. *obtusifolium* to a species and named it *H*. *obtusifolium* (Turn.) Brid. He argued his proposal with the following observations: “*caule subsimplici, foliis arcte imbricato—distichis concavis obtusis mediotenus uninerviis, thecae oblongae nutantis operculo e convexo rostrato*”, indicating that the described plant is characterized by, e.g., simple stems; with tightly overlapping, concave, obtuse leaves and alternate foliage; an oblong capsule, with a waving rostrum; and a convex lid. The species combination proposed by Bridel [7] is now obviously recognized as a homotypic synonym of *P*. *denticulatum* var. *obtusifolium* (Turner) Moore [3].

At the turn of the nineteenth and twentieth centuries, Stirton [14,15] distinguished two new species—*P*. *kinlayanum* Strit. and *P*. *annotinum* Stirt. He described the features of the former as: “elliptical leaves, and the cells very broad” that may resemble *P*. *denticulatum* var. *obtusifolium* but also many taxa of this genus. Elsewhere, Stirton [14,15] described *P. annotinum* as: “allied to *H*. *denticulatum*, but distinct in the curiously interrupted manner of growth of the branches; in the margins of the leaf reflexed almost to the apex”. In subsequent years Dixon [16] made *P*. *kinlayanum* and *P*. *annotinum* synonyms of *P. denticulatum* var. *obtusifolium* [3,12,16].

Amann [10] considered the names *P*. *obtusifolium* Brid. and *P*. *denticulatum* var. *donni* Lindb. as synonyms. Moreover, he pointed out some discrepancies in the nomenclature of this moss propounded by bryologists such as Lindberg [8], Dixon [16], Limpricht [17], Roth [18], Brotherus [19]. These authors reported the described taxon as var. *doni* [‘*Doni*’ in the protologue] or *donnii* (another orthographic equivalent of *donnianum*) or *P*. *denticulatum*. Amann [10], however, pointed out that it differs from the latter by inter alia “broadly ovate, very concave and curved leaves with a rounded, short apex”, which with these features refers to the plants currently named *P*. *denticulatum* var. *obtusifolium*. In the author’s opinion, this moss deserves to be distinguished as a species, as proposed by Bridel [7], Wahlenberg [5], and Mitten [9]. Currently, the first two cited names (*P*. *obtusifolium* Brid. and *P*. *denticulatum* var. *donni* Lindb.) are considered synonyms for *P*. *denticulatum* var. *obtusifolium* (Turn.) Moore [12,20].

In the mid-20th century, Koppe [21] used the name *P*. *denticulatum* f. *laticuspis* Koppe for *P*. *sylvaticum sensu* Mönkemeyer [22], which is now recognized as *P. denticulatum* var. *obtusifolium* [23], suggesting that the first name mentioned is also a synonym of the *P*. *denticulatum* var. *obtusifolium*.

In 1965, Ireland examined the types of *P*. *sandbergii* (PC0132604, PC0132605), stored in the Parisian herbarium, but, in a revision published four years later [24], he synonymized this and several other taxa with *P*. *denticulatum*, mentioning that these are only “environmental forms” of this species. However, in another article [25], he pointed out that this taxon requires detailed research. On the other hand, Wynns [3] indicated that this taxon is “evidently belonging to *P*. *denticulatum* var. *obtusifolium s*. *str*.”, but formal synonymization has not been carried out so far.

Currently, *P*. *denticulatum* var. *obtusifolium* is described as a glossy, pale to dark green plant [26]; stems 3 cm long [25], densely foliate, more or less julaceaous [20,25,27] or subjulaceous [20]; leaves almost symmetrical [25,27], asymmetrical, or symmetrical [26], ovate or widely ovate [20], strongly concave [25,26], strongly complanate, or imbricate [26], 1.4–1.6 mm long [28], 1.5–1.7 mm [20,29] or 1.5–2.0 mm long [25], and 0.8–0.9 mm [28] or 1 mm wide [20]; apex rounded or obtusely [20,25,26,27] and widely acuminate [20]; cell 90 μm long and 18 μm wide [30]; decurrencies composed of fewer inflated, quadrate or spherical cells [25] or the same as *P*. *denticulatum* [20]; and capsules straight and erect [25].

The data provided above indicate that the described variety differs from *P*. *denticulatum* with shorter julaceous stems (3 cm long) and almost symmetric, firmly concave, narrowly obtuse leaves up to 2 mm long. On the contrary, *P. denticulatum* has larger, rather flattened, acute and asymmetric leaves. *Plagiothecium denticulatum* var. *obtusifolium* has fewer inflated alar cells, while *P. denticulatum* has more inflated alar cells and inclined capsules [25]. This variety also differs from the species with its short, julaceous branches and obtuse leaf apex [29,31]. Thus, it is easy to see that the specialist literature lacks detailed data on even the basic, taxonomically significant features of this variety and differences between this taxon and *P*. *denticulatum*. Taxonomic features reported by many authors [3,20,25,26,27,29,32] indicate that *P*. *denticulatum* var. *obtusifolium* is currently quite variable.

Based on these observations and, above all, considering that the original material for this name has never been studied and described in detail, and, hence, no metric data on *P*. *denticulatum* var. *obtusifolium* currently exists, the aim of the present paper is to revise its original material and propose new synonyms for the analyzed taxon based on taxonomic revision of the original material of selected names in the genus *Plagiothecium*.

## 2. Results

### 2.1. Hypnum denticulatum var. obtusifolium (=P. denticulatum var. obtusifolium) Case

Turner [1] in the protologue does not cite any specimen or collection on the basis of which he described the new taxon; he only cites Figure 2 from Table 12 “T. 12. f. 2”, which illustrates selected features of *H*. *denticulatum* var. *obtusifolium* as well as those different from *H*. *denticulatum* (Figure 1 and Figure 3). Taking into account the above facts and according to Art. 9.1 of the *Shenzhen Code* [33], “A holotype of a name of a species or infraspecific taxon is the one specimen or illustration (…) either (*a*) indicated by the author(s) as the nomenclatural type or (*b*) used by the author(s) when no type was indicated”, it should be assumed that Figure 2 from Table 12, “T. 12. f. 2.” (Figure 3), is the holotype of the *H. denticulatum* var. *obtusifolium* name.

**Figure 4:**
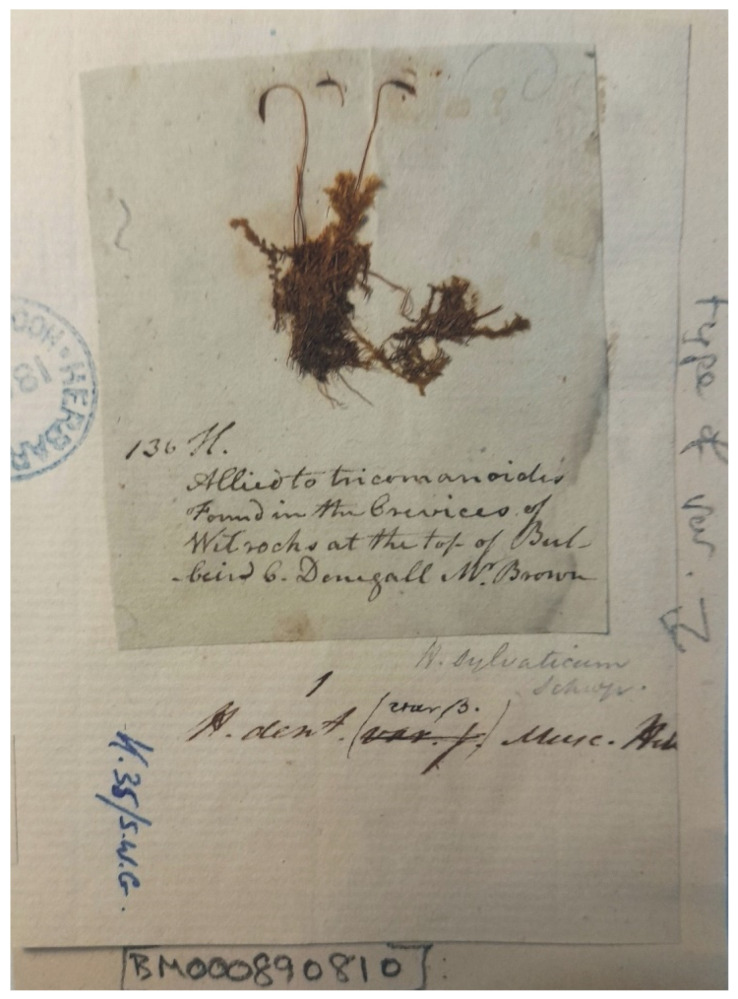
Specimen of *H*. *denticulatum* var. *obtusifolium* (BM000890810), photo by G.J. Wolski, Łódź, March 2022.

On the other hand, the specimen found in the BM herbarium (BM000890810) is not only not quoted in the protologue but also does not contain any date or signature of any researcher associated with the analyzed name (Figure 3 and Figure 4). Additionally, the description attached to the analyzed specimen, unfortunately, is only consistent with the diagnosis in a few respects, e.g., the same number “13.”, which is analogous to the number of this taxon in Turner book [1] and the mountain “Bulbein” where the described taxon was found. Thus, at the present stage, the specimen of *H*. *denticulatum* var. *obtusifolium* (BM000890810) stored at the Natural History Museum, London, England, is not a type specimen of this name; only with a high probability can it be indicated that it is the original collection on the basis of which Turner described the new taxon [1].

As mentioned above, the holotype of *H*. *denticulatum* var. *obtusifolium* (Figure 3) is a figure that shows the habit of this plant: julaceous stem; imbricate leaves; leaf (only a very general shape); shape; arrangement of the capsule; and the lid. However, these drawings are very simple and schematic, and the entire figure, from a taxonomic point of view, is devoid of many important features, which would allow one to clearly distinguish this taxon from other closely related species. It is missing, e.g., a detailed drawing of the leaf, with smooth and obtuse apex; cells’ dimensions; and the shape of decurrent cells.

However, due to the fact that the holotype does not include many taxonomically significant features, and taking into account Art. 9.9 of the *Shenzhen Code* “An epitype is a specimen or illustration selected to serve as an interpretative type when the holotype (…) is demonstrably ambiguous and cannot be critically identified for purposes of the precise application of the name to a taxon (…)”, a specimen recently found in the herbarium BM (BM000890810) should be designated as the epitype.

**Figure 5:**
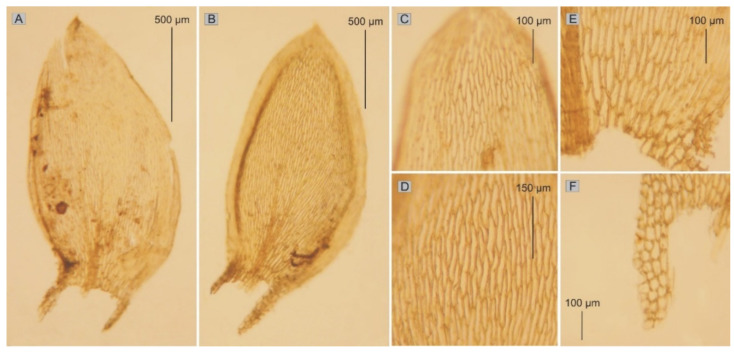
The most important taxonomic features of the epitype of *H*. *denticulatum* var. *obtusifolium*: (**A**,**B**) leaves; (**C**) cells from the top; (**D**) from the middle part; (**E**) from the lower part of the leaf; (**F**) decurrent cells (from the *H*. *denticulatum* var. *obtusifolium* BM000890810, photo by G.J. Wolski, Łódź, April 2022).

*Hypnum denticulatum* var. *obtusifolium* (=*Plagiothecium denticulatum* var. *obtusifolium sensu* *stricto*) is a small, soft plant with delicate metallic luster; stems are julaceous, 9–10 mm long (SL), rounded in cross section, with mean diameter 380–410 µm (SD); height and width of epidermal cells (HE, WE) are 8–15 µm (M 11.5) × 17–24 µm (M 20.5); height and width of peridermal cells (HP, WP) 18.5–64 µm (M 41.5) × 20–50 µm (M 35); densely foliate; leaves imbricate, not folded, concave, asymmetrical or slightly asymmetrical, ovate to elliptical; those from the middle of the stem are 1.5–2.2 mm (M 1.8) long (LL), and the width (LW) measured at the widest point is 0.80–1.2 mm (M 1.0); the apex obtusely apiculate; costae two, extending usually up to one-half of the leaf length, reaches (CL) 400–730 µm (M 555); the laminal cells length and width are very variable but depend on location: 43–125 (M 85) × 6–16.5 (M 11.25) μm at the apex (LC1, WC1); 64–140 (M 105) × 7–18 (M 11) μm at the midleaf (LC2, WC2); and 37–156 (M 86) × 8–33 (M 18) μm (LC3, WC3) at the base (Figure 5); due to the very wide cells, areolation is very loose; decurrency length (DL) is between 215 and 500 µm (M 330); alar cells (ACL, ACW) are rounded-rectangular 20–107 (M 48) × 14–41 (M 27.5) µm; setae maroon, 1.5–2.3 cm long (SPL), is gently twisted at the top; capsules are horizontal, 1.75–3.5 mm long (CPL, including operculum) and 0.5 mm wide (CPW) when dried; and operculum is about 0.75–1 mm long.

All of the above features, i.e., 40 quality and quantitative characteristics, made it possible to make redescription of the *Hypnum denticulatum* var. *obtusifolium* (=*Plagiothecium denticulatum* var. *obtusifolium sensu stricto*).

### 2.2. Plagiothecium sandbergii Case

**Figure 6:**
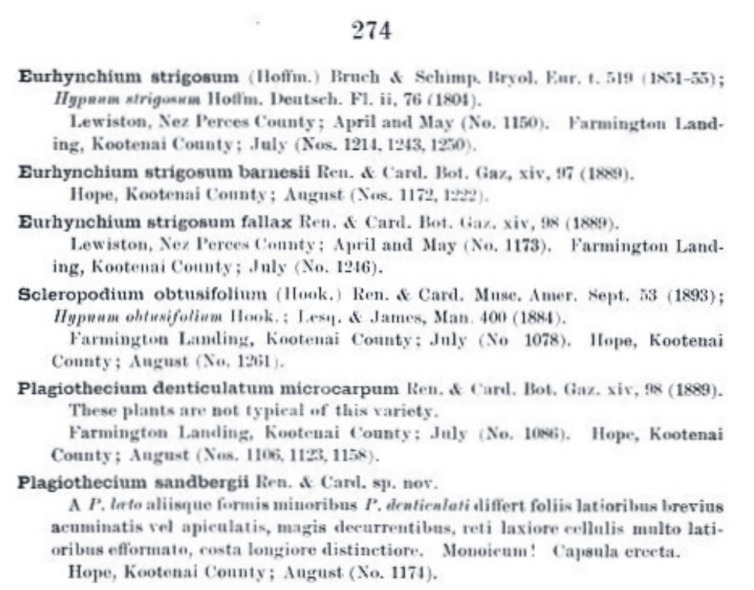
Diagnosis of *P*. *sandbergii* Ren. & Cardot [34].

Ferdinand François Gabriel Renauld and Jules Cardot, in *Contributions from the United States National Herbarium*, published *P*. *sandbergii* [34]. The authors noted that the taxon is smaller than *P*. *denticulatum*, has broader leaves, demonstrates cell areolation, and differs in apex. Despite the modest description, these features do indeed refer to the described taxon (Figure 6). In the protologue, the authors indicated that this material came from “Hope, Kootenai County” and is described as specimens “(No. 1174)”.

**Figure 7:**
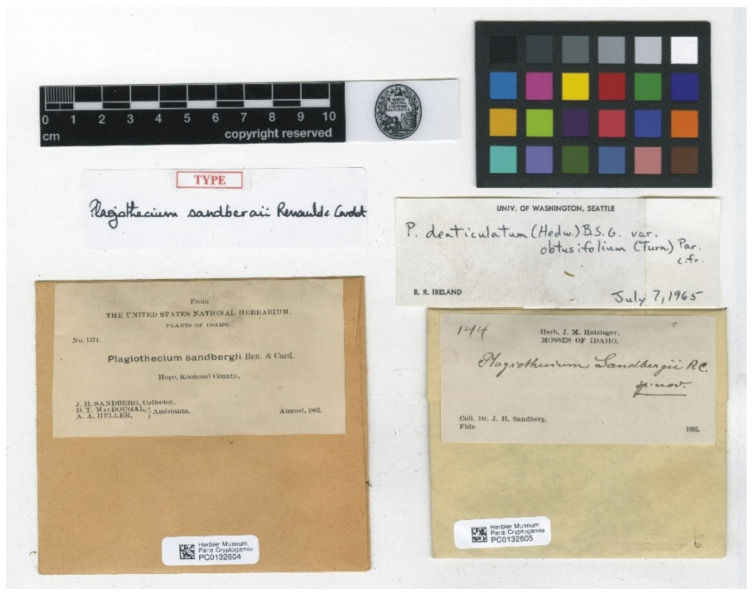
Types of *P*. *sandbergii* (PC0132604, PC0132605), from the herbarium PC website (https://science.mnhn.fr), downloaded 15 April 2022.

**Figure 8:**
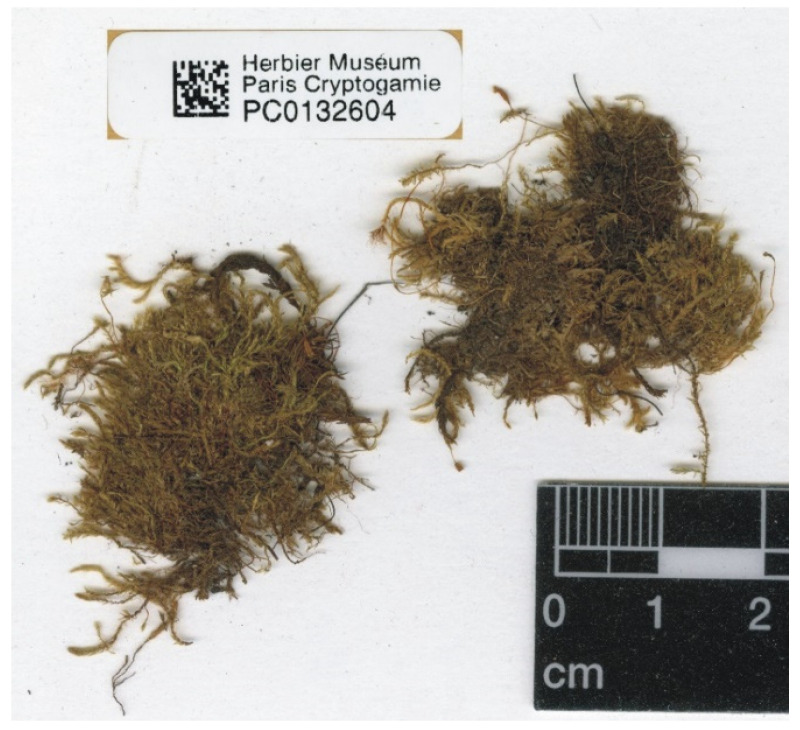
Turfs of *P*. *sandbergii*, specimen PC0132604. From the herbarium PC website (https://science.mnhn.fr), downloaded 15 April 2022.

**Figure 9:**
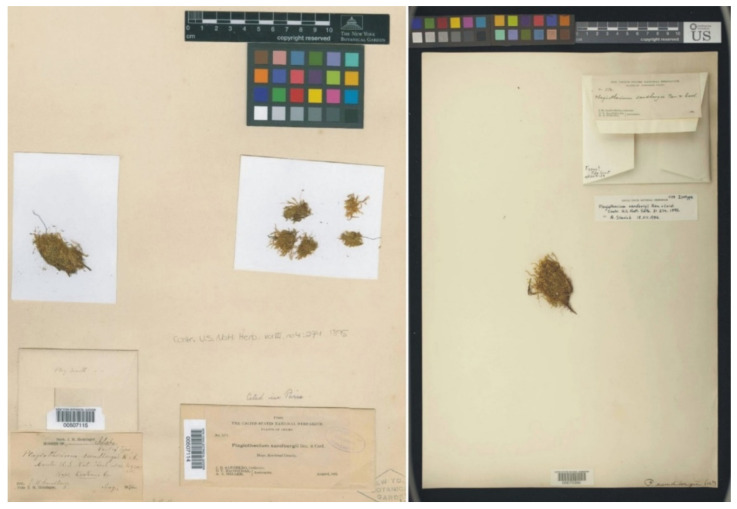
Two other syntypes of *P*. *sandbergii*. On the left, a sheet with specimens from the NY herbarium, on the right, a specimen deposited in the US herbarium (from https://bryophyteportal.org (accessed on 15 April 2022)).

Among the seven currently known “types” of *P*. *sandbergii* only four (PC0132604, US70396, NY507114, and FH220148) are signed as a sample “No. 1174“, and they are the only ones that can be considered syntypes of this species with certainty (Figure 7, Figure 8 and Figure 9). Considering the above, the fact that the specimen from the Muséum National d’Histoire Naturelle Paris, France (Herbarium PC) is characterized by a fairly large turf, and given Article 9.3 of the *Shenzhen Code* [33], quoted above, specimen PC0132604 from Herbarium PC should be designated as the lectotype of *Plagiothecium sandbergii* Renauld & Cardot (Figure 7 and Figure 8).

**Figure 10:**
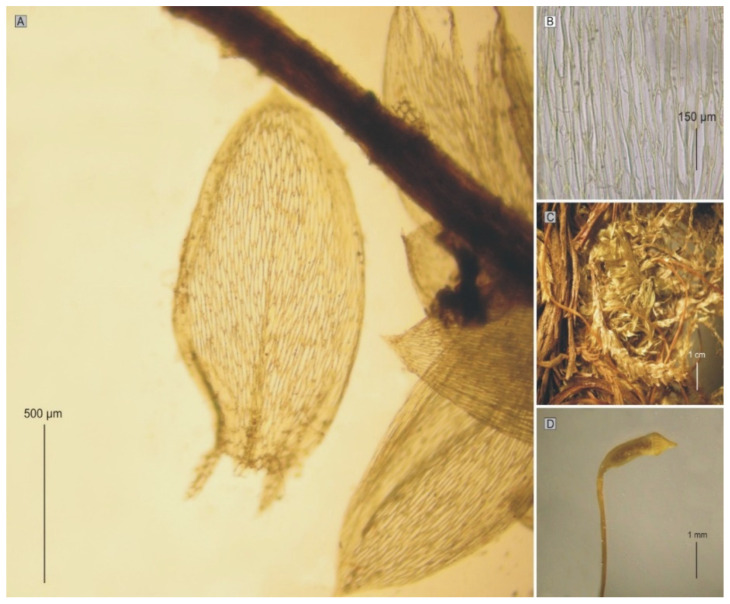
The most important taxonomic features of *P*. *sandbergii*: (**A**) leaves; (**B**) cells from the middle part of the leaf; (**C**) stems; (**D**) sporophyte (photo from PC0132604, PC0132605 by G.J. Wolski, Paris, November 2021).

*Plagiothecium sandbergii* is a small, light green plant with characteristic metallic luster; stem is creeping, julaceous, rounded in cross section, with diameter 230 µm (SD); leaves are imbricate, concave and gently asymmetrical, not folded, ovate to elliptical; those from the middle of the stem are 1.0–1.3 mm long (LL), and width (LW) is measured at the widest point 0.5–0.58 mm (M 0.56); the apex is obtusely apiculate; costae two, extending usually up to half leaf length, reaches (CL) 290–460 µm (M 380); the cells linear, length, and width are very variable but depend on location, 25–90 (M 60) × 7–16 (M 10) μm at the apex (LC1, WC1); 58–130 (M 92) × 6–21 (M 11) μm at the mid-leaf (LC2, WC2); and 18–81 (M 44) × 6–20 (M 12) μm (LC3, WC3) at the base (Figure 10); due to the very wide cells, cell areolation is clearly loose; decurrency length (DL) is between 102 and 213 µm (M 151); alar cells (ACL, ACW) are rounded-rectangular 20–75 (M 35) × 6–18 (M 11.5) µm; setae maroon, 1.5–1.8 cm long (SPL), is twisted; capsules are horizontal, 2.0–2.5 mm long (CPL, including operculum) and 0.5 mm wide (CPW) when dried; and operculum is about 0.5–0.75 mm long.

### 2.3. Plagiothecium denticulatum var. auritum Case

**Figure 11:**
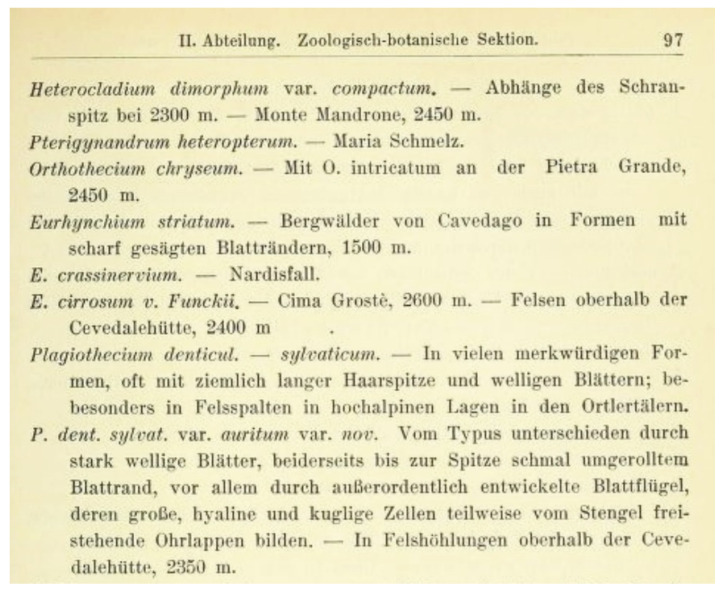
Diagnosis of *P*. *denticulatum* var. *auritum* (Kern 1914).

Friedrich Kern [35] published *P*. *denticulatum* var. *auritum* in the *Jahresbericht der Schlesischen Gesellschaft für Vaterländische Cultur*. In the diagnosis, he indicates only selected features of the described species, indicating that it is distinguished from *P*. *denticulatum* by strongly folded leaves and well-developed decurrences, with hyaline and spherical cells. The author added that it is observed in the hollows of rocks. Kern [35] did not provide any collection or material that was used to describe the new variety or any drawing of the new taxon (Figure 11).

**Figure 12:**
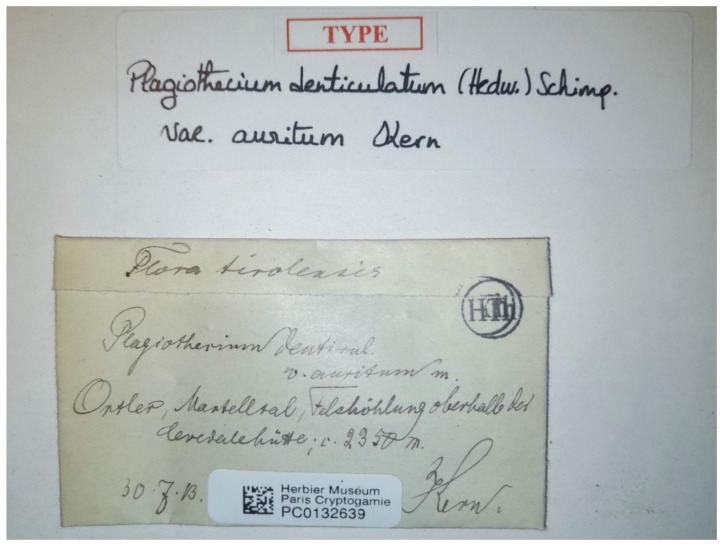
Type of *P. denticulatum* var. *auritum* (PC0132639), photo by G.J. Wolski, Paris, November 2021.

**Figure 13:**
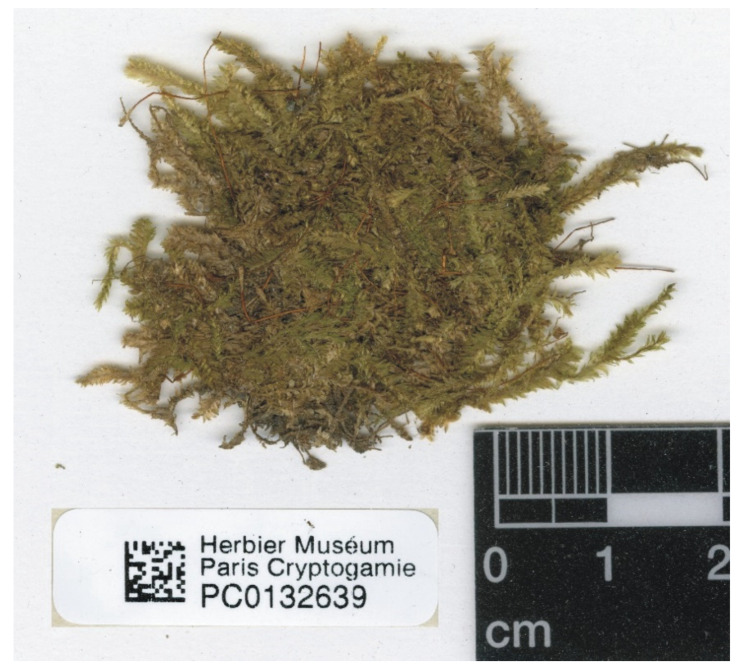
Turf of *P*. *denticulatum* var. *auritum* (specimen PC0132639). From the herbarium PC website (https://science.mnhn.fr), downloaded 15 April 2022.

A specimen of *P*. *denticulatum* var. *auritum* (PC0132639) was found at the Muséum National d’Histoire Naturelle in Paris; this is the only material currently known that could be used as a base for the description by Kern [35]. Considering the above and according to Article 9.3 of the *Shenzhen Code* [33], cited above, specimen PC0132639 from the Herbarium PC should be designated as the lectotype of *Plagiothecium denticulatum* var. *auritum* Kern (Figure 12 and Figure 13).

**Figure 14:**
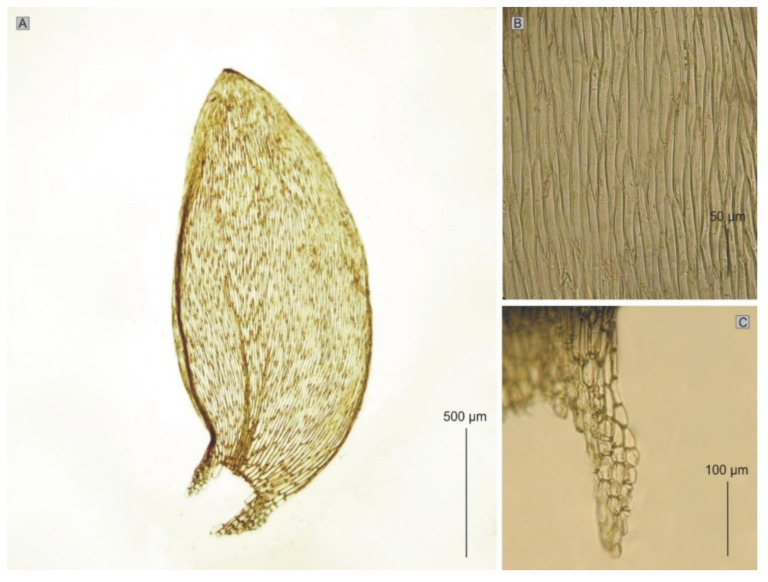
The most important taxonomic features of *P. denticulatum* var. *auritum*: (**A**) leaves; (**B**) cells from the middle part of leaves; (**C**) decurrent cells (photo from PC0132639 by G.J. Wolski, Paris, November 2021).

*Plagiothecium denticulatum* var. *auritum* is a small, light green plant with metallic luster; stem creeping, 2.0–2.5 cm, rounded in cross-section; leaves not folded, concave, ovate and asymmetrical, those from the middle of the stem 1.4–1.8 mm (M 1.60) long (LL), and 0.7–1 mm (M 0.85) wide (LW), when measured at the widest point (Figure 14); the apex is obtuse or obtusely apiculate; costae two, extending usually up to half leaf length, reaches (CL) 426–632 µm (M 497); cell is laminal, and dimensions are highly variable and depend on location: 30–95 (M 62) × 4–14 (M 9) μm at apex (LC1, WC1); 50–140 (M 101) × 6–20 (M 10) μm at midleaf (LC2, WC2); and 30–165 (M 69) × 9–19 (M 13) μm (LC3, WC3) at base, due to the very wide cells, cell areolation is clearly loose; decurrency length (DL) is between 128 and 260 µm (M 181); and alar cells (ACL, ACW) are rounded-rectangular 15–50 (M 32) × 7–19 (M 13) µm.

**Figure 15:**
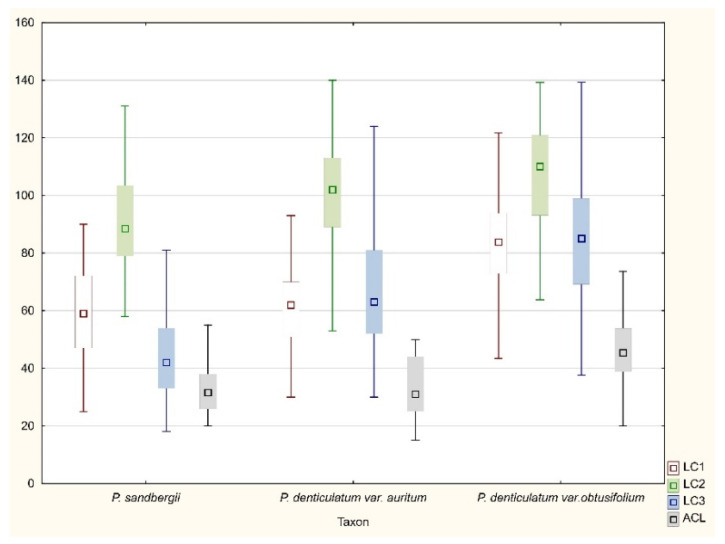
The range of variability of the examined features. Explanation: LC1, length of cells from the top; LC2, the middle part; LC3, from the lower part of the leaf; ACL, length of alar cells (length in μm).

**Figure 16:**
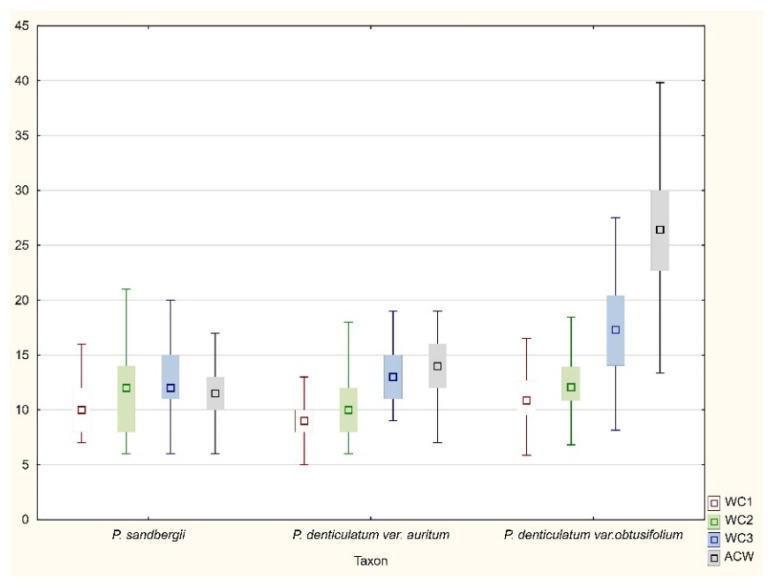
The variability range of the examined features. Explanation: WC1, width of cells from the top; WC2, the middle part; WC3, from the lower part of the leaf; ACW, width of alar cells (length in μm).

**Figure 17:**
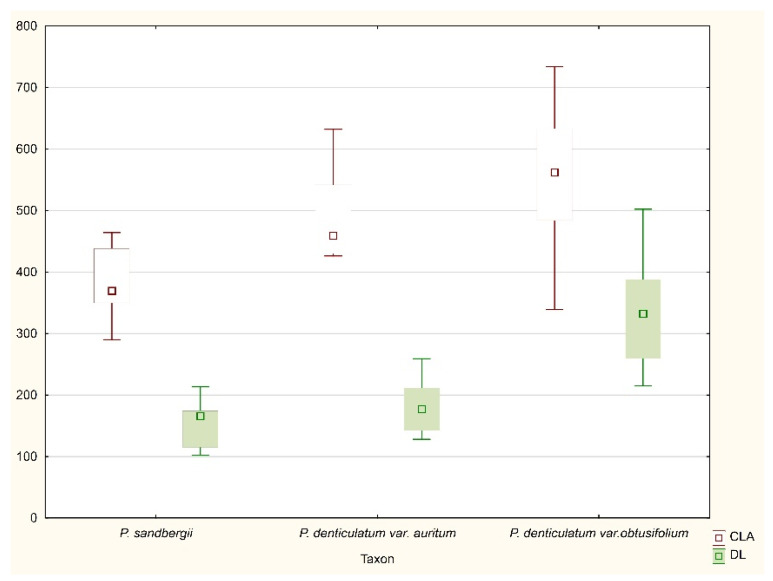
The range of variability of the examined features. Explanation: CL, average costae length; DL, decurrency length (length in μm).

All tested specimens did not differ considerably with regard to any of the most important taxonomic features (Figure 15, Figure 16 and Figure 17). Therefore, two examined taxa (*P*. *denticulatum* var. *auritum*, *P*. *sandbergii*) did not differ from *H*. *denticulatum* var. *obtusifolium* (=*P*. *denticulatum* var. *obtusifolium*), as indicated not only by the above descriptions, but also by the analysis of the ranges of variability of individual features (Figure 15, Figure 16 and Figure 17). Therefore, based on these results and all the facts cited above, we propose that currently separate taxa, *P*. *sandbergii* and *P*. *denticulatum* var. *auritum*, should be considered new synonyms of *P*. *denticulatum* var. *obtusifolium*.

### 2.4. Description of Plagiothecium denticulatum var. obtusifolium sensu lato

All examind specimens were characterized by their light green color, fine metallic luster, and relatively small size; stem length (SL) 0.9–2.5 cm, in cross section rounded with a mean diameter (SD) of 230–410 μm; height and width of epidermal cells (HE, WE) is 8–15 µm × 17–24 µm; height and width of peridermal cells (HP, WP) is 18.5–64 µm × 20–50 µm; leaves are julaceous, very concave, ovate-elliptical, gently asymmetrical, and not folded; the length of leaves (LL) is from 1.0 to 2.2 mm, and width (LW) 0.5–1.2 mm; the apex is obtusely apiculate; costae two, extending usually up to one-half of the leaf length, ranges from 290–730 μm (CL) (M 512); the cells are linear, length and width are very variable but depend on location: 25–125 × 4–16 μm at the apex (LC1, WC1); 50–140 × 6–21 (M 13.5) μm at midleaf (LC2, WC2); and 18–165 × 6–33 μm (LC3, WC3) toward insertion; due to the very broad cells, cell areolation is clearly loose; broad decurrencies are with length (DL) between 102 and 500 µm; alar cells are rounded-rectangular 15–105 × 6–40 µm (ACL, ACW); setae maroon, 1.5–2.3 cm long (SPL), gently twisted at the top; capsules are horizontal, 1.75–3.5 mm long including operculum and 0.5 mm wide (CPL, CPW) when dried; and operculum (OL) is about 0.75–1 mm long (Figure 15, Figure 16 and Figure 17).

## 3. Discussion

In the second half of the 20th century, some bryologists abandoned attempts to distinguish all of the varieties or forms described above related to *P*. *denticulatum* var. *obtusifolium*. Many of them have mentioned the variant in the context of an ecotype, with a slightly different appearance from *P*. *denticulatum sensu lato*, caused by habitat conditions. One of the first to propound this theory was Ireland [24,25]. He pointed out that the julaceous forms called *P. denticulatum* var. *obtusifolium* by Greene [28] and others are just environmental forms of *P. denticulatum* restricted to mountaintops and cliffs. Furthermore, Ireland [24,25] claims that any *P. denticulatum* plant is capable of producing julaceous stems under proper environmental conditions; this was later supported by Anderson et al. [36], Düll [37], Ignatov and Afonina [38], and Deng-ke and Ireland [39]. As a result, all the abovementioned authors considered *P*. *denticulatum* var. *obtusifolium* as a synonym of *P*. *denticulatum*. However, current genetic studies [3] confirm the validity of distinguishing this name as a separate taxon, and there are no lower-rank taxa within it [12,26].

So far, *H*. *denticulatum* var. *obtusifolium*, now known as *P*. *denticulatum* var. *obtusifolium*, has not been studied in detail or received a detailed description. A review of the literature confirms that this taxon is very superficially described, and many of its taxonomic features are not recorded [20,25,27,28,29,30].

The specimen found in the herbarium BM, described there as “type”, was used to designate the epitype of this name. The analysis of the studied material made it possible to describe many new features that have not been described in the literature so far [20,25,27,28,29,30], including stem diameter (SD); epidermal cell height and width (HE, WE); peridermal cell height and width (HP, WP); costa length (CL); dimensions of leaf tip and base cells (LC1, WC1, LC3, WC3); length of decurrencies (DL); alar cell length and width (ACL, ACW); seta length (SPL); and dimensions of capsules (CPL, CPW) and operculum (OL).

Studies of *H*. *denticulatum* var. *obtusifolium* specimen also revealed significant differences between the analyzed features and what has been published in the literature to date. Smith [26] reports that the leaves of this taxon are strongly complanate or imbricate, and, while they are indeed imbricate, neither the examined material nor the accompanying figure [1] (Figure 3) indicates that they are strongly or even delicately complanate. Furthermore, it should be noted that the julaceous, imbricate leaves of the taxon are one of its most important and taxonomically significant features, distinguishing it from other closely related species [1,12].

Analysis of leaf dimensions of the *H*. *denticulatum* var. *obtusifolium* reveals a greater variation (1.48–2.2 × 0.82–1.2 mm) than indicated in the literature [20,25,28,29]. Our present findings indicate that the length of the leaves is within the lower limit reported by Greene [28] and exceeds the upper limit reported by Ireland [25], while their width is within the lower limit reported by Greene [28] and exceeds the upper limit reported by Iwatsuki [20].

Similar observations concern the range of variation in the length and width of the cells of the central part of the leaf. Only Jedlička [30] provides relevant data, reporting that they reach 90 μm in length and 18 μm in width. However, our present findings indicate greater variation among the cells of *H*. *denticulatum* var. *obtusifolium*, both in terms of length (64–139 μm) and width (7–18 μm). The relative lack of data from the literature is surprising, as the cells in the middle part of the leaf are among the most important taxonomic features of the genus *Plagiothecium*, allowing individual species to be distinguished from each other based on a given section [12,20,27,40,41].

Another interesting incongruency between our present data and previous findings is highlighted when reviewing Ireland [25], who notes that decurrencies are composed of fewer inflated, quadrate, or spherical cells, while the material of *H*. *denticulatum* var. *obtusifolium* is characterized neither by quadrate cells nor by their small number. In this taxon, as Iwatsuki [20] writes, “decurrencies do not differ from those in *P*. *denticulatum*” and are composed of many inflated and spherical cells. Currently, many authors [3,12,23,40] indicate that, while the shape of decurrent cells accurately differentiates the taxa of the described genus into sections, this is not related to interspecific and intraspecific differentiation. Ongoing research supports this assertion.

Ireland [25] indicates that the capsules of this taxon are “straight and erect”. This is particularly interesting as it contradicts ongoing research. A detailed analysis of the *H*. *denticulatum* var. *obtusifolium* specimen (BM 000890810), the description (Figure 1), and the holotype of this name (Figure 3) indicate that the material examined is unambiguously characterized by an inclined or at least oblique, but never a “straight and erect”, capsule; this is also the case for the entire *P*. *denticulatum* complex.

As reported by Greene [28], *Plagiothecium* species are usually collected sterile and sporophytes are not often recorded. Furthermore, as Wolski [42,43] points out, the most important taxonomic features of this genus concern the qualitative and quantitative features of the gametophyte. Hence, few data are available on the sporophytes of the described variety. As such, there may be some discrepancies in capsule orientation, as in the aforementioned paper by Ireland [25]. It is also known that, like many other mosses, *Plagiothecium* species form heterogeneous turfs, sometimes with several similar species in one sample. Distinguishing these species may be a challenge, especially when the taxa are closely related and quite similar, as in the case of *P*. *laetum* Schimp. and *P*. *curvifolium* Schlieph. ex Limpr., which often grow side by side and occupy a similar habitat [12].

In the second half of the 20th century, in North America and Asia [20,24,25], *P*. *laetum* and *P*. *curvifolium* were considered synonyms rather than separate species. Despite this, they differ in many features, one of which is the orientation of the capsule, which is upright in *P*. *laetum* but oblique in *P*. *curvifolium*. Current DNA analyses [3,40] clearly support the validity of distinguishing these taxa and treating them as separate species.

Hence, *P. denticulatum* var. *obtusifolium* appears to have a horizontal capsule, indicating that Ireland [25] may have analyzed mixed material or described another similar taxon, with a “straight and erect” capsule. This is supported by the fact that a species quite similar to the analyzed variety, characterized by distinctly julaceous, imbricate, concave, symmetrical leaves, and erect capsules, was recently described from North America (Aleutians, Alaska). This taxon, *P*. *schofieldii* G.J. Wolski & W.R. Buck [44] is recorded in nonforested areas of the Aleutian Islands and, due to its rather similar morphological appearance, could have been confused with *P*. *denticulatum* var. *obtusifolium* in the second half of the 20th century.

Although the data from the literature demonstrate a number of divergencies with our current findings, some convergencies were also observed. Our findings confirm that the anayzed specimen of *H*. *denticulatum* var. *obtusifolium* is a small plant, as described by Iwatsuki [20] and Ireland [25]; it is characterized by a delicate metallic luster [26] with dense and more or less julaceous foliage, as indicated by previous researchers [20,25,27]. This study also confirms observations made by scientists in Europe [26,27], North America [25], and Asia [20] that the leaves of this taxon are symmetrical or gently asymmetrical, distinctly strongly concave, mostly ovate, and imbricate [26]; and the apex is rounded or obtuse [20,25,26,27].

Taken together, these features indicate some degree of similarity with some species from Southern and Northern Hemisphere taxa. Wynns [3] indicated that *P*. *denticulatum* var. *obtusifolium* is similar to a taxon recorded in the Southern Hemisphere—*Plagiothecium lamprostachys* (Hampe) A.Jaeger, based on its julaceous or braided stems and concave ovate leaves.

However, some smaller, prostrate specimens of *P*. *denticulatum* var. *obtusifolium* from the Pacific Northwest look similar to *Plagiothecium pacificum* [3]. *Plagiothecium denticulatum* var. *obtusifolium* is also similar to *Plagiothecium schofieldii* mentioned above [44]. Furthermore, Wynns [3] identifies several more taxa that are morphologically similar to *P. denticulatum* var. *obtusifolium*, including: *Plagiothecium cochleatum* Dixon, with its well-developed decurrencies, and *Plagiothecium denticulatum* var. *auritum* Kern. Whereas, Ukrainskaya [29] characterized *P. denticulatum* var. *obtusifolium* as an intermediate form between *Plagiothecium berggrenianum* Frisvoll and *P. denticulatum* var. *denticulatum*. However, to date, detailed studies of the original collections and types of taxa mentioned above have not been investigated.

Interestingly, Wynns [3] notes that “in the Northern Hemisphere the name *P. denticulatum* var. *obtusifolium* has also been widely applied to non-julaceous plants with plicate leaves, wide cells and strongly and broadly recurved margins”. The author also uses the name *Plagiothecium denticulatum* var. *bullulae* Grout for such plants from western North America and Greenland, while Eurasian plants are listed under “*P. denticulatum* var. *obtusifolium in herb*”.

Even more intriguing, Smith [26] provides some significantly different findings, i.e., that *P*. *denticulatum* var. *obtusifolium* is also found in New Zealand, Tasmania, or even in the southern parts of South America. This is probably due to a mistake in the determination of the analyzed material, since several species may resemble the described variety. One such example is the abovementioned *P*. *lamprostachys*, which by its julaceous or braided stems and concave, ovate leaves could be mistaken for the described taxon. However, to be sure of this conclusion, of course, one first should revise the materials that this author analyzed.

Many authors indicate that *P*. *denticulatum* var. *obtusifolium* is associated with mountainous areas, at high latitudes in boreal localities [3,20,26,28,45]. However, recent analyses of the distribution of the genus *Plagiothecium* in Eurasia [12] report that it is more commonly found in Europe and certain Asian countries, including China, Russia, Nepal, Japan, and Iran, rather than in the boreal zone.

Wynns [3] mentions *P*. *denticulatum* var. *auritum*, stating only that “Kern’s original materials should be compared with European collections listed under *P*. *denticulatum* var. *obtusifolium*”. However, the author did not study any types for this taxon but analyzed some specimens from the original collection of *P. sandbergii*, mentioning only that these specimens are “belonging to *P*. *denticulatum* var. *obtusifolium sensu stricto*”. The present study, enriched by analysis of the remaining specimens from the original *P*. *sandbergii* collection, confirms these observations. Additionally, they unambiguously indicate that both *P*. *denticulatum* var. *auritum* and *P*. *sandbergii* match the holotype and epitype of *H*. *denticulatum* var. *obtusifolium* in terms of both gametophyte and sporophyte characteristics. Thus, these taxa were proposed as new synonyms of *P*. *denticulatum* var. *obtusifolium*.

### Taxonomic Treatment

***Plagiothecium denticulatum* var. *obtusifolium*** (Turner) Moore, Proceedings of the Royal Irish Academy 1: 424 (1873); *Hypnum denticulatum* var. *obtusifolium* Turner, Muscologiae Hibernicae Spicilegium 146, T. 12, f. 2 (1804); *Plagiothecium obtusifolium* (Turner) J.J. Amann, Mémoire de la Société Vaudoise des Sciences Naturelles 3: 61 (1928). ***Holotype***: Figure 2, Tabela 12 “T. 12, f. 2” (Turner 1804: 237) (Figure 3). ***Epitype*** (*designated here*): [Ireland], in summo montis *Bulbein* jugo, ab oculatissimo *D*. *Brown* lectam, benigne communicavit D. Templeton, BM000890810! (Figure 5).
*=Plagiothecium sandbergii* Ren. & Cardot, Contributions from the United States National Herbarium, 3: 274 (1895), **syn. nov. *Lectotype*** (designate here): U.S.A., Idaho, Kootenai County, Hope, *J*.*H*. *Sandberg*, *D*.*T*. *Macdougal*, *A*.*A*. *Heller 1174*, August 1892 (PC0132604!); ***isolectotypes***: NY507114!, available online; US70396!, available online; FH220148. Additional original material from locus classicus (not signed “No. 1174”): NY507115!, available online. Additional Sandberg’s material, potentially from locus classicus: PC0132605! Additional Sandberg’s material: FH220147 (https://kiki.huh.harvard.edu/databases/specimen_search.php?mode=details&id=144580 (accessed on 12 August 2022)).
*=Plagiothecium denticulatum* var. *auritum* Kern, Jahresbericht der Schlesischen Gesellschaft für Vaterländische Cultur 91 Abt. 2b. 97 (1914), **syn. nov. *Lectotype*** (designate here): [Italy] South Tirol, Ortler, Martelltal, in Felshöhlungen oberhalb der Cevedalehütte, *F*. *Kern s.n.*, 2350 m, 30 July 1913, herb. H. Thériot (PC0132639!).

## 4. Materials and Methods

### 4.1. Herbarium Materials

In diagnosis, Turner [1] does not cite any specimen or collection as a type. However, during a revision at the Natural History Museum, London, UK (herbarium BM) a specimen of *Hypnum denticulatum* var. *obtusifolium* (BM000890810) was found, which is labeled in this herbarium as a “type”. This specimen was received as a loan for a detailed study and analysis (Figure 4). In 1867, this material was incorporated into Hooker’s herbarium, and on the left is visible a part of the seal “Herbarium Hookerianum”. Additionally, on the sheet of this specimen are many handwritten annotations, including the rewriting of “var. γ” to “var. β”. However, the analysis of the description of this specimen and the diagnosis of *H*. *denticulatum* var. *obtusifolium* confirm that it is var. γ, and the right specimen for planned analyses.

In addition, types of other species of the studied genus, *P*. *sandbergii* Ren. & Cardot (PC0132604, PC0132605) (Figure 7 and Figure 8) and *P*. *denticulatum* var. *auritum* Kern (PC0132639) (Figure 12 and Figure 13), from the Muséum National d’Histoire Naturelle, Paris, France (Herbarium PC), were examined.

### 4.2. Measurement Method

**Figure 18:**
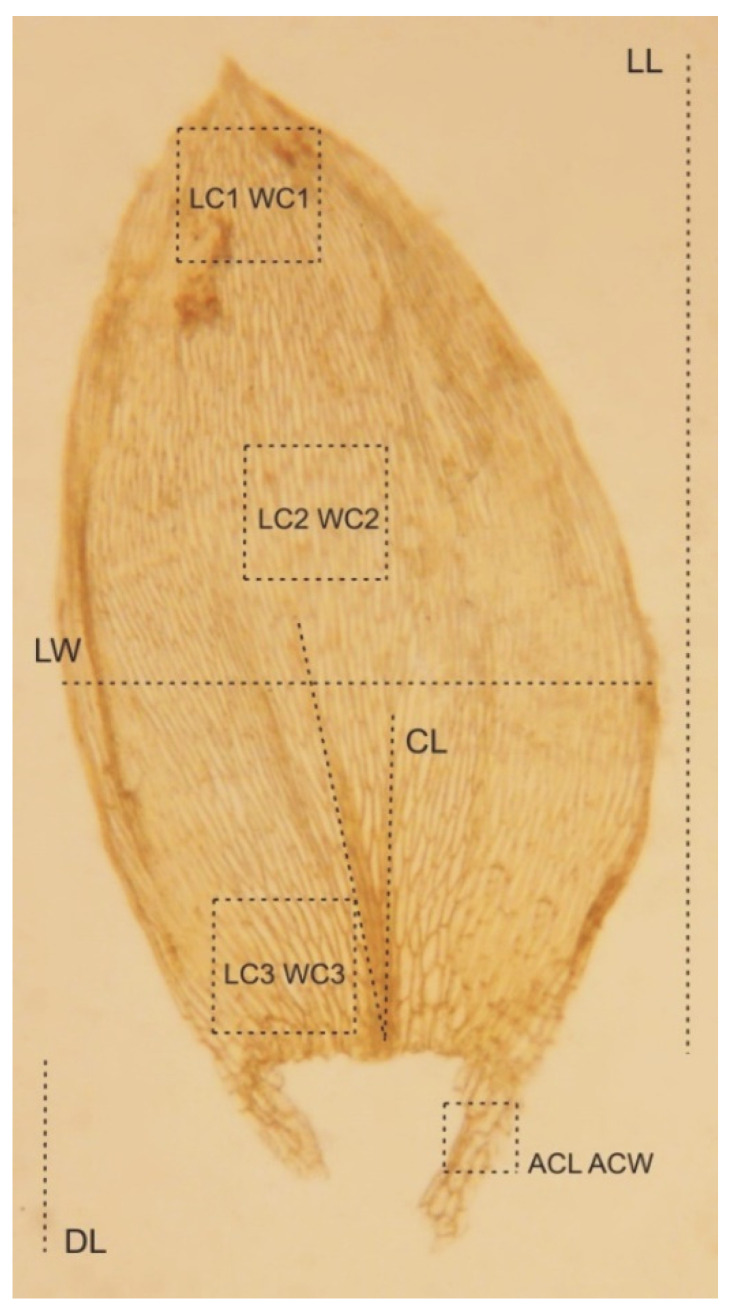
Location of the characteristics studied on the leaf. Illustration from the original materials of *H*. *denticulatum* var. *obtusifolium* (BM000890810) by G.J. Wolski, April 2022.

Forty qualitative and quantitative characteristics were analyzed, e.g., stem length (SL) and stem diameter (SD); height and width of epidermal cells (HE, WE); height and width of peridermal cells (HP, WP); lengths and widths of leaf (LL, WL); average costae length (CL); length and width cell from the top (LC1, WC1), middle part of the leaf (LC2, WC2), and lower part of the leaf (CL3, WC3) (Figure 18); length and width of alar cell (ACL, ACW); length and widths of decurrencies (DL, DW); sporophyte length (SPL); capsule length (with operculum) (CPL); and operculum length (OL). In the case of qualitative traits, leaf shape (LS), concavity (LCO), and leaves symmetry (LSY); apex shape (AS); and alar cell shape (ACS) were analyzed.

In the case of the leaf length and width (LL, LW); length and width of cells from the top (LC1, WC1), the middle part (LC2, WC2), and from the lower part of the leaf (LC3, WC3); the length and width of alar cells (ACL, ACW); average costae length (CL;) and the decurrency length (DL) were included in the mathematical analysis. Therefore, to visualize the differences or similarities between the groups, box plot diagrams are presented. All analyses were performed using STATISTICA software (version 13.3.7).

All specimens of *P*. *sandbergii* included in the statistical analyses were collected by J.H. Sandberg. Therefore, since the material of this species was represented by two separate specimens (PC0132604, PC0132605) (Figure 7 and Figure 8), any data obtained for them were summed and referenced jointly as representing a single taxon.

## Data Availability

All source data are contained in the manuscript, while the raw data are available at https://drive.google.com/drive/folders/1Th_WZV_eV5pWYPqv5WDXxl4ar0HEpEWA?usp=sharing (13 August 2022).
